# Establishing an effective gene knockdown system using cultured cells of the model fish medaka (*Oryzias latipes*)

**DOI:** 10.1093/biomethods/bpac011

**Published:** 2022-05-17

**Authors:** Kosuke Zenke, Yasushi Okinaka

**Affiliations:** Graduate School of Integrated Sciences for Life, Hiroshima University, Higashihiroshima, Hiroshima 739-8528, Japan; Graduate School of Integrated Sciences for Life, Hiroshima University, Higashihiroshima, Hiroshima 739-8528, Japan

**Keywords:** medaka, gene knockdown, siRNA, model animal, transfection

## Abstract

In spite of the growing attention given to medaka (*Oryzias latipes*) as an excellent vertebrate model, an effective gene knockdown system has not yet been established using cultured cells of this fish species. In this study, a gene knockdown system using short interfering RNA (siRNA) in medaka cell lines was established through the optimization of transfection conditions. By extensive screening of several medaka cell lines and transfection reagents, OLHNI-2 cells and X-tremeGENE siRNA Transfection Reagent were selected as the best combination to achieve high transfection efficiency of siRNA without cytotoxic effect. Knockdown conditions were then refined using the endogenous heat shock protein 90 (Hsp90) genes as the siRNA targets. Among the parameters tested, cell density, serum concentration in the culture medium, and duration of transfection improved knockdown efficiency, where the target mRNA in cells transfected with each of the siRNAs was reduced from 12.0% to 26.7% of the control level. Our results indicate that the established knockdown system using siRNA is a promising tool for functional analysis of medaka genes *in vitro*.

## Introduction

Medaka (*Oryzias latipes*) is an excellent vertebrate model that is used in various study areas including development, genetics, environmental toxicology, and human disease [1–4]. In addition to its preferred characteristics as a model animal, such as small body size, high fecundity, tolerance to a wide range of environmental conditions, and ease of rearing, the availability of several inbred lines and the enormous genetic diversity between those lines [5] are distinct features of medaka not found in other model fish species. Moreover, whole-genome sequencing [6] and the establishment of transgenesis, mutagenesis, and genome editing systems have been accomplished in medaka [7–10].

To date, most knockdown studies in medaka have been conducted using developing embryos (e.g. [11–13]). One important genetic tool that remains to be developed in medaka is an effective knockdown system using cultured cells. A cultured cell-based knockdown system is suitable for examining a large number of genes and quantitative analyses compared with an embryo-based strategy that includes laborious steps of microinjection.

Short interfering RNA (siRNA) is a commonly used knockdown tool. When introduced into eukaryotic cells, siRNA degrades specific mRNA via the RNA interference (RNAi) machinery [14]. siRNA is cost-effective and can be designed to target any sites of mRNA compared with morpholino antisense oligos, another widely used knockdown tool [15]. siRNA-based knockdown studies have been extensively performed using embryos (e.g. [16–18]) and cultured cells (e.g. [19]) in zebrafish, a well-recognized model fish. Similar knockdown studies using cultured cells were also reported in some other ordinary fish species including barramundi (*Lates calcarifer*) [20], carp (*Cyprinus carpio*) [21], Chinook salmon (*Oncorhynchus tshawytscha*) [22], fathead minnow (*Pimephales promelas*) [23], grouper (*Epinephelus coioides*) [24, 25], Japanese flounder (*Paralichthys olivaceus*) [26], marine medaka (*Oryzias melastigma*) [27], and sea perch (*Lateolabrax japonicus*) [28]. However, the efficiencies of these knockdown systems using cultured fish cells were generally low compared with those using cultured mammalian cells. In this study, we established an effective siRNA-based knockdown system using cultured medaka cells by optimizing conditions for siRNA transfection as well as by testing some medaka cell lines. Our new knockdown system would improve the efficiency of the genetic research in medaka and in turn should attract more scientists to choose medaka as an experimental material.

## Materials and methods

### Medaka cell lines

The OLHE-131 cell line was purchased from RIKEN BRC Cell Bank. OLHNI-2, OLKaga-e1, OLHdrR-e3, OLCAB-e21, and OLCAB-e31 [29] were provided by H. Mitani. All the medaka cell lines were cultured at 30°C in Leibovitz’s L-15 medium (L-15) (Invitrogen, Carlsbad, CA, USA) supplemented with 15% fetal bovine serum (FBS) (Nissui, Tokyo, Japan). At 16 h before transfection, medaka cells were seeded on 12- and 24-well cell culture plates (Sumitomo Bakelite, Tokyo, Japan) at the cell density of 3.2 and 1.6 × 10^5^ cells per well, respectively, which achieved about 80% confluency at the time of transfection.

### siRNAs

The fluorescently labeled control siRNA (BLOCK-iT Alexa Fluor Red Fluorescent Oligo) was purchased from Invitrogen. The siRNAs targeting heat shock protein 90 (Hsp90) genes with Stealth modification were designed using BLOCK-iT RNAi Designer and purchased from Invitrogen. The Stealth modification is the chemical modification to siRNA, which enhances the potency and stability of siRNA and reduces the occurrence of off-target effect. The target sequence of each siRNA is listed in Table 1. Stealth RNAi GFP reporter control (Invitrogen), which targets enhanced green fluorescent protein, was used as the negative control siRNA (siRGFP). All the siRNAs were dissolved at the concentration of 20 µM in RNase-free distilled water and stored at −20°C until use.

**Table 1:** siRNAs used in this study

siRNA	Location^a^	Sequence (5′–3′)
siRHsp90α1-1	213-228	GCTGAAGATTGAAGTCAGACCTGAT
siRHsp90α1-2	459-484	CGAGCAGTATATCTGGGAATCTGCA
siRHsp90α1-3	536-551	GCACTAAAGTGATCCTCCACCTCAA
siRHsp90α1-4	1012-1036	AGAGCTGCCTTTGACCTCTTCGAAA
siRHsp90α1-5	1859-1884	TGACAGCCAAGAAGCATCTGGAGAT
siRHsp90α2-1	536-561	GAACCAAAGTGATCCTCCACCTGAA
siRHsp90α2-2	898-923	GAGGATCACCTGGCTGTCAAGCATT
siRHsp90α2-3	552-577	CCACCTGAAAGAAGATCAGTCAGAA
siRHsp90α2-4	684-709	CGACGAGGACAAACCTGAGATTGAG
siRHsp90α2-5	1805-1830	CGACTATGGGATACATGGCTGCTAA
siRHsp90β-1	581-606	AGAAGAGGGTCAAAGAGATCGTGAA
siRHsp90β-2	851-876	CCATCTGGACCAGAAACCCTGATGA
siRHsp90β-3	1093-1118	GAGCTCATCCCAGAGTACCTGAACT
siRHsp90β-4	1581-1606	CAAGAACCTGGTTTCTGTCACCAAA
siRHsp90β-5	1682-1707	GCAAGCTCATGAAAGAGATTCTGGA

a Adenine residues of the start codons are designated as “1.”

### Transfection of the fluorescently labeled siRNA

Medaka cells seeded on 24-well plates were transfected with the fluorescently labeled control siRNA at the final concentration of 80 nM using each of the various transfection reagents listed in Table 2 according to the manufacturer’s instructions, except that medium containing the transfection complex was replaced with fresh L-15 medium containing 15% FBS at 6 h after transfection. The volumes of transfection reagents were determined according to the manufacturer’s recommendations for initial optimization experiments. Transfection efficiency of fluorescently labeled siRNA and cytotoxic effects on the transfected cells were examined at 24 h after transfection using fluorescence and light microscopic observations, respectively.

**Table 2:** Transfection reagents used in this study

Transfection reagent (supplier)	Volume (μl/well)^a^
RiboJuice siRNA Transfection Reagent (Novagen, Darmstadt, Germany)	2.0
Lullaby-siRNA transfection reagent (OZ Biosciences, Marseille, France)	4.0
INTERFERin (Polyplus, Illkirch, France)	3.0
jetPRIME (Polyplus)	3.0
HiPerFect Transfection Reagent (Qiagen)	3.0
Fugene HD Transfection Reagent (Roche Diagnostics)	1.5
X-tremeGene siRNA Transfection Reagent (Roche diagnostics)	2.5
MultiFectam (Promega)	12.5
HilyMax (Dojindo, Kumamoto, Japan)	3.0
TransIT-TKO Transfection Reagent (Mirus, Madison, WI, USA)	2.5
Lipofectamine 2000 (Invitrogen)	1.0

a The reagents were used at the indicated volumes.

### Transfection of the siRNAs targeting Hsp90 genes

OLHNI-2 cells seeded on 12-well plates were transfected with the siRNAs targeting Hsp90 genes using X-tremeGENE siRNA Transfection Reagent (Roche Diagnostics, Basel, Switzerland) according to the manufacturer’s instructions with some modifications. Then, 4 µl of the siRNA stock solution (20 µM) and 5 µl of X-tremeGENE siRNA Transfection Reagent were separately diluted in 100 µl Opti-MEM-I (Invitrogen). The diluted siRNA and transfection reagent was then combined and incubated for 20 min at room temperature. Finally, the transfection complex was diluted with 800 µl L-15 medium containing 15% FBS and was applied to cells after removal of culture medium. The final concentration of siRNA became 80 nM. After incubation for 6 h, the medium was replaced with 1 ml fresh L-15 medium containing 15% FBS and the cells were further incubated for 42 h to be sacrificed for total RNA isolation for quantitative real-time RT-PCR.

To optimize transfection conditions, various modifications to the initial conditions described above were tested independently: (i) the final concentrations of siRNA were adjusted at 40, 80, and 120 nM; (ii) 2.5, 5, and 10 µl per well of the transfection reagent were used; (iii) cells were seeded at the densities of 1.6, 2.0, 2.4, and 3.2 × 10^5^ cells per well, which achieved about 40%, 50%, 60%, and 80% confluency at the time of transfection, respectively; (iv) the transfection complex was diluted with 800 µl L-15 medium containing 0%, 5%, and 15% FBS; (v) the medium containing the transfection complex was replaced or diluted with 1 ml fresh L-15 medium containing 15% FBS after incubation for 6 h; and (vi) cells were incubated at 25°C, 30°C, and 35°C during transfection.

### Transfection of siRNAs using the optimized transfection conditions

At 16 h before transfection, OLHNI-2 cells were seeded on 12-well culture plates at the density of 2.0 × 10^5^ cells per well, which achieved 50% confluency at the time of transfection. Then, 4 µl of the siRNA stock solution (20 µM) and 5 µl of X-tremeGENE siRNA Transfection Reagent were separately diluted in 100 µl Opti-MEM-I. The diluted siRNA and transfection reagent were then combined and incubated for 20 min at room temperature. Finally, the transfection complex was diluted with 800 µl L-15 medium containing 5% FBS and was applied to cells after removal of the culture medium. The final concentration of siRNA became 80 nM. After incubation for 6 h, 1 ml fresh L-15 medium containing 15% FBS was applied to the cells, which were further incubated for 42 h until total RNA isolation for quantitative real-time RT-PCR analysis. Cells were cultured at 30°C during the entire experiment period.

### RNA isolation and reverse transcription

Total RNA was isolated from cells using the acid guanidinium thiocyanate–phenol–chloroform method [30] and treated with RQ1 RNase-free DNase (Promega, Madison, WI, USA), followed by purification using the RNeasy mini RNA isolation kit (Qiagen, Valencia, CA, USA). To obtain complementary DNA (cDNA), 0.5 µg purified total RNA was incubated with 0.5 µl oligo dT primer (20 µM; Operon, Tokyo, Japan) at 70°C for 10 min and further incubated at 42°C for 60 min supplemented with 2 µl 5× RT reaction buffer (Takara, Otsu, Japan), 2 µl dNTP mix (10 mM each, Takara), 100 units M-MLV reverse transcriptase (RNase H–, Takara), and 10 units RNase inhibitor (Toyobo, Osaka, Japan) in a final reaction volume of 10 µl. The synthesized cDNA samples were diluted 10 times with double-distilled water and stored at –20°C until use.

### Quantitative real-time RT-PCR

Quantitative real-time RT-PCR was performed using Thunderbird SYBR qPCR Mix (Toyobo) and the Chromo4 Real-Time PCR Detection System (Bio-Rad, Hercules, CA, USA). The primer sets used to detect medaka Hsp90 and β-actin genes are listed in Table 3. The reaction mixture for PCR contained 10 µl Thunderbird SYBR qPCR Mix, 0.5 µl of each primer (10 µM), 3 µl of the cDNA sample, and 6 µl double-distilled water in a final reaction volume of 20 µl. The amplification procedures included one cycle of 3 min at 95°C, followed by 40 cycles of 30 s at 95°C and 30 s at 60°C. Using melting curve analysis, the relative mRNA expression level was calculated by the standard curve method using the β-actin mRNA expression level as the control. Data are presented as the ratios (%) against those of control cells receiving no transfection.

**Table 3:** Detection primer sets used in this study

Name^a^	Sequence (5′–3′)
Hsp90-α1-F	ATGTCATGGAGGAGGAGGTG
Hsp90-α1-R	GGAGATGAGCTCTCGAAGGA

Hsp90-α2-F	GATCAGTCAGAATACCTGGAG
Hsp90-α2-R	TTGTCCTCGTCGTCACTCAC

Hsp90-β-F	GAGTACATTGAGGAGAAGAGG
Hsp90-β-R	TCCTCACCTTCCTCCTTGG

β-Actin-F	GGGAGAAGATGACCCAGATC
β-Actin-R	ACCAGAGTCCATGACGATAC

a F, forward primer and R, reverse primer.

### Data analysis

All data were analyzed with one-way or two-way ANOVA as appropriate, followed by multiple comparisons using Tukey’s HSD test.

## Results

### Screening of medaka cell lines and transfection reagents

For the initial step of optimization, we screened out the optimal combination of medaka cell line and transfection reagent that achieved the highest transfection efficiency of siRNA and the lowest cytotoxic effect. Representative images showing the uptake of fluorescently labeled siRNA are presented in Supplementary Fig. S1. Among the 11 transfection reagents (Table 2), X-tremeGENE siRNA Transfection Reagent gave the highest transfection efficiencies in all of the medaka cell lines tested (Table 4). Some other transfection reagents (MultiFectam, HilyMax, and Lipofectamine 2000) showed similarly high transfection efficiencies in OLHE-131 and OLHNI-2 cells. When using these four highly competent transfection reagents, siRNA uptake was slightly greater in OLHNI-2 than in OLHE-131. Although a cytotoxic effect was not observed in most of the combinations of cell lines and transfection reagents, cells transfected with siRNA using Lipofectamine 2000 showed severe-to-moderate cell death (Table 4). Based on these results, we used X-tremeGENE siRNA Transfection Reagent and the OLHNI-2 cell line for further optimization experiments.

**Table 4:** Transfection of medaka cell lines with fluorescently labeled siRNA using various transfection reagents

Transfection reagent^a^	1	2	3	4	5	6	7	8	9	10	11
siRNA uptake^b^											
OLHE-131	++	+	–	++	–	+	+++	+++	+++	–	+++
OLHNI-2	++	++	+	++	–	+	+++	+++	+++	–	+++
OLKaga-e1	+	+	–	++	–	+	+++	++	+	–	–
OLHdrR-e3	+	+	–	++	–	+	+++	++	–	–	–
OLCAB-e21	++	+	–	++	–	+	+++	++	–	–	–
OLCAB-e31	++	+	–	++	–	+	+++	++	–	–	–
Cytotoxic effect^c^											
OLHE-131	–	–	–	–	–	–	–	–	–	–	+
OLHNI-2	–	–	–	–	–	–	–	–	–	–	+
OLKaga-e1	–	–	–	–	–	–	–	–	–	–	+
OLHdrR-e3	–	–	–	–	–	–	–	+	++	–	+++
OLCAB-e21	–	–	–	–	–	–	–	+	++	++	+++
OLCAB-e31	–	–	–	–	–	–	–	+	++	–	+++

a 1, RiboJuice siRNA Transfection Reagent; 2, Lullaby-siRNA transfection reagent; 3, INTERFERin; 4, jetPRIME; 5, HiPerFect Transfection Reagent; 6, Fugene HD Transfection Reagent; 7, X-tremeGene siRNA Transfection Reagent; 8, MultiFectam; 9, HilyMax; 10, TransIT-TKO Transfection Reagent; and 11, Lipofectamine 2000.

b Amount of cells uptaking siRNA; +++ (>80%), ++ (40∼80%), + (10∼40%), and – (<10%).

c Amount of cells exhibiting cytotoxicity; +++ (>80%), ++ (40∼80%), + (10∼40%), and – (<10%).

### Knockdown efficiency under a preoptimized condition

**Figure 1:**
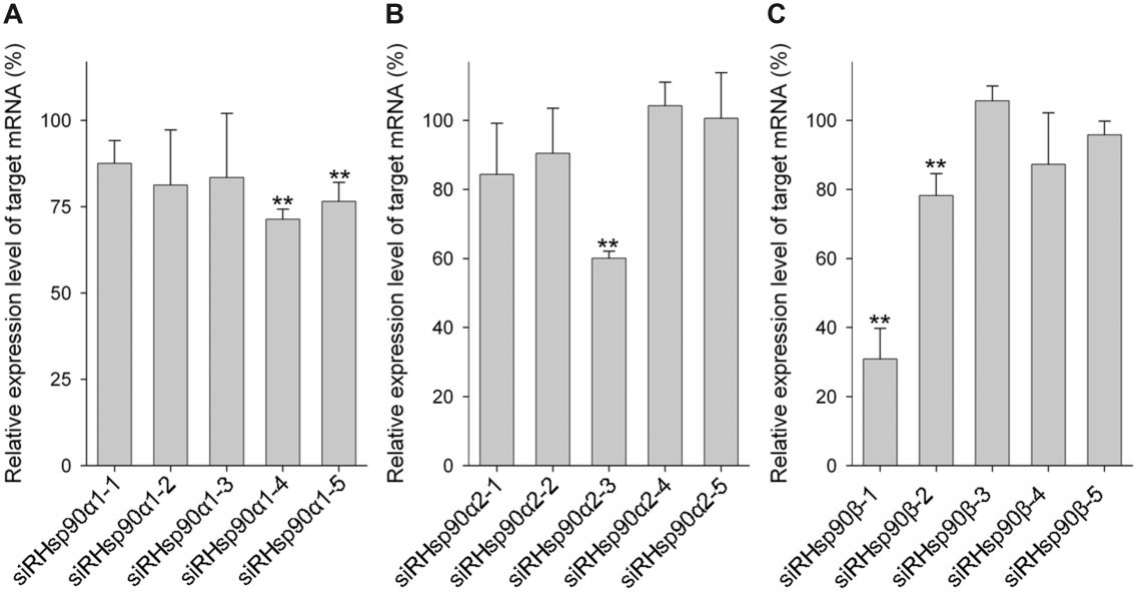
Knockdown of medaka Hsp90 genes. OLHNI-2 cells seeded on 12-well culture plates were transfected with each of the five siRNAs targeting (**A**) Hsp90α1, (**B**) Hsp90α2, or (**C**) Hsp90β at the final concentration of 80 nM using 5 µl X-tremeGENE siRNA Transfection Reagent. At 48 h after transfection, total RNA was isolated from the cells and the relative expression level of target mRNA was determined by real-time RT-PCR analysis. Data are presented as mean values of three independent experiments with standard deviations. **P < 0.01 compared with the control.

We first examined the knockdown efficiency of siRNA using OLHNI-2 cells and X-tremeGENE siRNA Transfection Reagent according to the supplier’s instructions. In this experiment, Hsp90 genes were selected as the knockdown targets since Hsp90 mRNAs are markedly abundant in eukaryotic cells [31] and are likely to be difficult to knock down without optimizing conditions. Among the 15 siRNAs targeting Hsp90α1, Hsp90α2, or Hsp90β genes (five siRNAs per gene), siRHsp90β-1 reduced the level of target mRNA (Hsp90β) down to 30.9% compared with the control (Fig. 1). OLHNI-2 cells transfected with some of the other siRNAs also showed slight to moderate reductions of the target mRNAs, ranging from about 60% to 80% of the control levels.

### Optimization of transfection conditions

To optimize transfection conditions, we tested various transfection parameters using siRHsp90β-1, which exhibited the highest knockdown efficiency among the 15 siRNAs under the preoptimized condition (Fig. 1).

#### (i) Effects of siRNA concentration and quantity of the transfection reagent on knockdown efficiency

**Figure 2:**
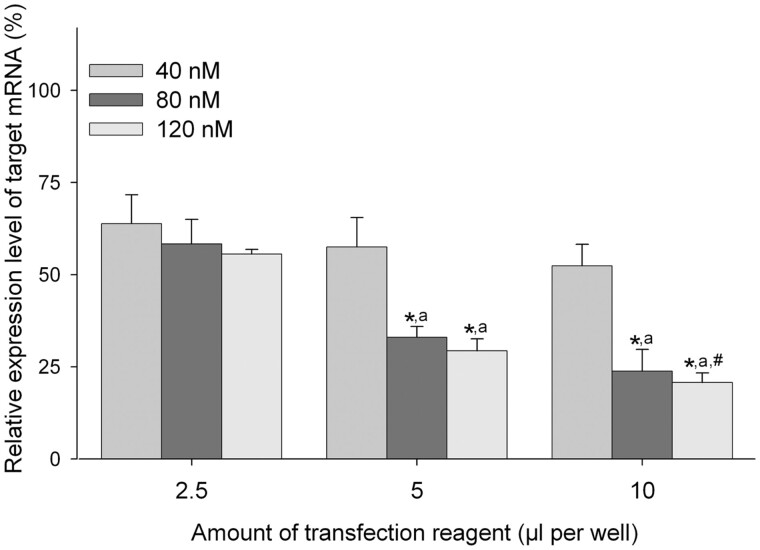
Effects of siRNA concentration and quantity of the transfection reagent on knockdown efficiency. OLHNI-2 cells seeded on 12-well culture plates were transfected with the siRNA siRHsp90β-1 at the different final concentrations using different quantities of X-tremeGENE siRNA Transfection Reagent. At 48 h after transfection, total RNA was isolated from the cells and the relative expression level of target mRNA was determined by real-time RT-PCR analysis. Data are presented as mean values of triplicate wells with standard deviations. *P < 0.01 compared with cells transfected with 40 nM siRNA using the same quantity of the transfection reagent; ^a^*P* < 0.01 compared with cells transfected with the same final concentration of siRNA using 2.5 µl of the transfection reagent; and ^#^*P* < 0.05 compared with cells transfected with 80 nM siRNA using 10 µl of the transfection reagent.

As shown in Fig. 2, reduction of target mRNAs occurred depending on the siRNA concentration and quantity of the transfection reagent. When OLHNI-2 cells were transfected with siRNA using 5 or 10 µl transfection reagent per well, the target mRNA was reduced significantly in cells transfected with 80 or 120 nM siRNA compared with cells transfected with 40 nM siRNA. Furthermore, knockdown efficiencies obtained using 10 µl transfection reagent were higher than those using 5 µl transfection reagent. The highest reduction of the target mRNA, corresponding to 20.4% of the control value, was observed in cells transfected with 120 nM siRNA using 10 µl of the transfection reagent, although approximately 50% of the transfected cells died. Similar cytotoxicity was observed in cells transfected with 80 nM siRNA using 10 µl of transfection reagent. Thus, to avoid such cytotoxic effects and to use a minimal siRNA concentration, we carried out transfection using 80 nM siRNA and 5 µl of the transfection reagent for further study.

#### (ii) Effect of cell density on knockdown efficiency

**Figure 3:**
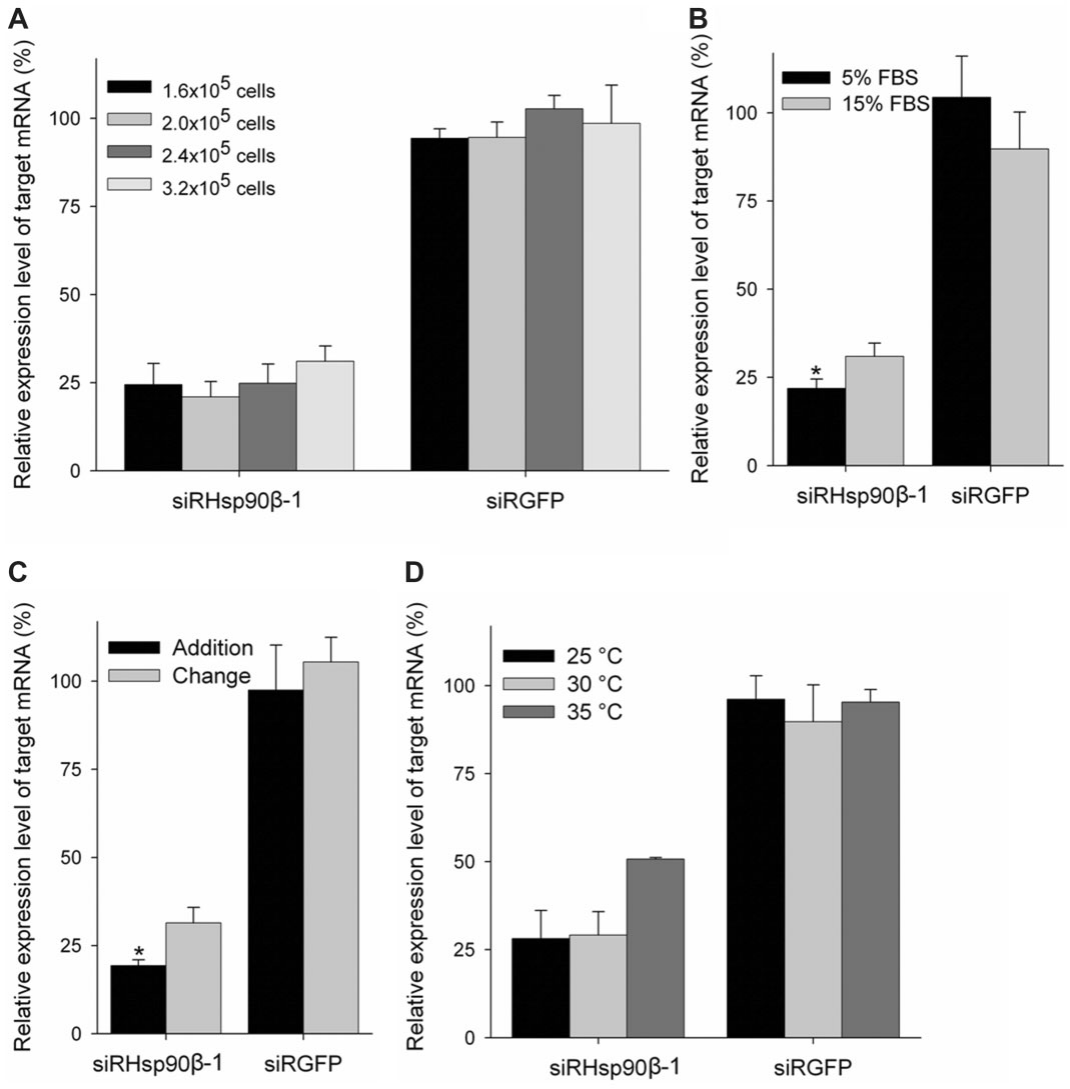
Optimization of transfection conditions. OLHNI-2 cells were transfected with the siRNA siRHsp90β-1 using the most effective transfection conditions shown in Fig. 2 with the modifications described below. (**A**) Cells were seeded at the cell density of 1.6, 2.0, 2.4, and 3.2 × 10^5^ cells per well. (**B**) Transfection complex was diluted with 800 µl L-15 medium containing 5% or 15% FBS before addition to cells. (**C**) The medium containing the transfection complex was replaced with 1 ml fresh L-15 medium supplemented with 15% FBS (change) or was diluted by applying 1 ml of the medium (addition). (**D**) Cells were incubated at 25°C, 30°C, and 35°C during transfection. The siRNA targeting an EGFP gene (siRGFP) served as the negative control. At 48 h after transfection, total RNA was isolated from the cells and the relative expression level of target mRNA was determined by real-time RT-PCR analysis. Data are presented as mean values of triplicate wells with standard deviations. *P < 0.05 compared with the control.

With the reduction of cell density from 3.2 × 10^5^ to 2.0 × 10^5^ cells per well of a 12-well culture plate, knockdown efficiency was improved slightly (Fig. 3A). The highest reduction of target mRNA to 21.0% compared with the control was observed when cells were seeded at the cell density of 2.0 × 10^5^ cells per well. Cells transfected with the negative control siRNA (siRGFP) exhibited no reduction of the target mRNA (Hsp90β) throughout this study (Fig. 3A).

#### (iii) Effect of serum concentrations on knockdown efficiency

When the serum concentration in the culture medium was kept at 5% during the first 6 h after transfection, knockdown efficiency was significantly improved without any cytotoxic effects (Fig. 3B). Cells that were cultured in the serum-free medium during the first 6 h after transfection died almost completely. The levels of the target mRNA in cells transfected under low (5%) and normal (15%) serum concentration were reduced to 19.7% and 31.0% compared with the control, respectively.

#### (iv) Effect of incubation time on knockdown efficiency

When cells were cultured in the medium containing the transfection complex for longer than 6 h, severe cytotoxic effect was observed. Thus, we added the fresh L-15 medium containing 15% FBS to the cells at 6 h after transfection to reduce the concentration of the transfection complex and avoid the occurrence of cytotoxic effect. The level of the target mRNA in the cells subjected to addition of the fresh medium was significantly reduced without cytotoxic effect compared with those subjected to complete exchange of the medium (19.3% and 31.4% compared with the control, respectively) (Fig. 3C).

#### (v) Effect of temperature on knockdown efficiency

We tested different culture temperatures during transfection to improve knockdown efficiency. However, there was no difference in the efficiency between the low (25°C) and normal (30°C) temperatures (Fig. 3D). Knockdown efficiency was even worse at 35°C (50.4% compared with the control), the temperature at which the cell growth rate was much higher than at 25°C or 30°C.

### Knockdown efficiency under the optimized condition

**Figure 4:**
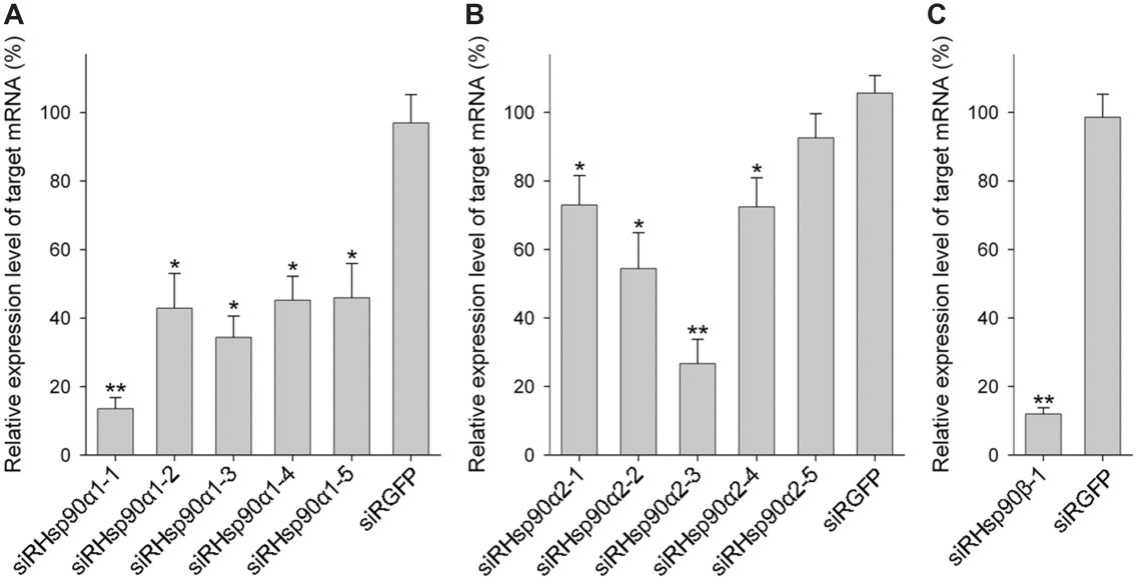
Reanalysis of knockdown efficiencies using optimized transfection conditions. OLHNI-2 cells were transfected with each of the five siRNAs targeting (**A**) Hsp90α1, (**B**) Hsp90α2, or (**C**) Hsp90β using the transfection conditions optimized in Fig. 3. The siRNA targeting an EGFP gene (siRGFP) served as the negative control. At 48 h after transfection, total RNA was isolated from the cells and the relative expression level of target mRNA was determined by real-time RT-PCR analysis. Data are presented as mean values of triplicate wells with standard deviations. *P < 0.05, **P < 0.01 compared with the control.

To provide proof of concept, most of the siRNAs shown in Fig. 1 were reexamined under the optimized transfection conditions. Generally, knockdown efficiencies were greatly improved (Fig. 4) compared with those under the preoptimized condition (Fig. 1). In particular, siRHsp90α1-1 and siRHsp90β-1 reduced the levels of the target mRNA expression to 13.6% and 12.0%, respectively, compared with the control. Similarly, siRHsp90α2-3 also exhibited relatively high knockdown efficiency as evidenced by the reduced level (26.7%) of the target mRNA.

## Discussion

In this study, we successfully established an siRNA-based gene knockdown system using cultured cells of medaka, a well-studied model vertebrate. With this system, Hsp90 mRNAs as the trial targets were efficiently degraded even though they are extremely abundant in eukaryotic cells. In our recent study, this optimized method was successfully applied to knock down Hsp70 genes in medaka to assess their roles as host factors in fish virus infection [32]. This *in vitro* genetic tool would facilitate gene function study in medaka, allowing us to perform large-scale screenings and quantitative analyses of genes of interest.siRNA is recognized as one of the most common genetic tools to study gene functions in mammalian cells, likely because commercially available transfection reagents and algorithms for designing siRNA are optimized for use for mammalian cell lines. Our first obstacle in establishing an effective knockdown system using medaka cell lines was the low transfection efficiency of siRNA. By examining the combinations of 6 cell lines and 11 transfection reagents, only X-tremeGENE siRNA Transfection Reagent was shown to deliver siRNA with more than 80% efficiency into all of the medaka cell lines tested. The transfection reagents tested in this study contain cationic lipids as the major functional molecules. Their functions to deliver siRNA into cells are based on the interactions between positively charged siRNA–lipid complexes (lipoplexes) and the negatively charged cell surface. Lipoplexes are then introduced into cells by endocytosis and siRNA is released into cytoplasm. Since lipid and fatty acid components in the cellular membranes differ between fish and mammals [33], transfection reagents supplied by commercial venders would not necessarily be applicable to fish cultured cells including medaka. X-tremeGENE siRNA Transfection Reagent, which was effective for all the medaka cells tested, might have a broader spectrum beyond species than the other transfection reagents used in this study. Remarkably, this transfection reagent also caused no cytotoxic effect in all the medaka cell lines tested, unless the reagent was treated at an extremely high concentration.

In this study, the siRNAs targeting Hsp90 genes were designed according to the instructions of the supplier, Invitrogen, which guarantees that two out of three siRNAs, designed using its algorithm, give a reduction of more than 70% of the target mRNA. However, only 3 of the 11 siRNAs tested in this study achieved this even under the optimized transfection conditions. We recently carried out similar knockdown experiments for another medaka gene using siRNA purchased from another company. In this case, only one of the four siRNAs tested gave a reduction of more than 70% of the target mRNA even though the expression level of this gene in the control cells was much lower than those of Hsp90 genes (Supplementary Fig. S2). These results suggest that the algorithms for designing siRNA so far available are optimized for mammals, but not for fish. This possibility is supported by the fact that knockdown levels in other fish species are similar to our results [20, 23, 24, 26, 27] and are lower than those in mammals [34, 35]. Otherwise, molecular structures of commonly used siRNAs might not be compatible with the siRNA-based silencing machinery in fish, which therefore leads to undesirably low siRNA activities in fish cells. Bohle *et al.* [36] demonstrated that transfection of the fish cells (CHSE-214) with chemically synthesized 27/25-nucleotide-long dsRNAs (DsiRNAs) with a concentration as low as 1 nM knocked down efficiently virus gene expression. In this system, DsiRNAs were cleavaged into 21-nucleotide-long siRNAs as the substrate for Dicer. Collectively, these results suggest that commonly used siRNAs cannot activate effectively the RNAi pathway in fish. To confirm this idea, further study to clarify the RNAi machinery in fish is needed.

Several parameters for transfection are known to influence greatly the results of knockdown experiments using siRNA [37, 38]. There is no universal protocol that is applicable to all kinds of cells. In this study, we examined the effects of six different parameters for transfection on knockdown efficiency. Of these parameters tested, the siRNA concentration and quantity of the transfection reagent most significantly affected the knockdown efficiency, where the combination of the highest doses of siRNA (among 40, 80, and 120 nM) and transfection reagent (among 2.5, 5, and 10 µl/well of a 12-well plate) showed the highest knockdown efficiency, although it was accompanied by moderate cytotoxic effects. Such high-dose treatment is likely to induce cytotoxicity by inhibiting microRNA pathways [39, 40] or by nonspecific alteration of several gene expressions [41]. Although it is obvious that knockdown efficiency was positively related to the siRNA concentration and quantity of the transfection reagent in our experimental settings, empirical optimization is needed in future individual studies to determine these two parameters which maximize the knockdown efficiency with minimal adverse effect.

Other culturing conditions also affected the knockdown efficiency in this study. Generally, functions of transfection reagents are disturbed by the components of serum [42, 43] with some exceptions [44]. In this study, low concentrations of serum in the culture medium improved the knockdown efficiency, suggesting that the X-tremeGENE siRNA Transfection Reagent was sensitive to serum. The reduction of serum concentration during transfection (15% before transfection, 5% during the first 6 h, and 10% during the remaining 42 h) is thought to contribute to the prolonged stabilization of lipoplexes in the culture medium. The stabilized lipoplex then allows cells to take up siRNA for a prolonged period, resulting in the improvement of knockdown efficiency. The prolonged duration of transfection is also suggested to improve the knockdown efficiency for a similar reason, where the culture medium containing lipoplex was not changed completely but diluted by fresh medium to reduce the cytotoxic effect.

In this study, the knockdown efficiency was improved by reducing cell density while the efficiency was negatively affected by increasing the culture temperature (35°C) above normal (30°C), which extensively promoted cell division. These observations suggest that the amount of siRNA taken up per cell might also influence the knockdown of target genes. In fact, previous studies have reported that dilution of siRNA by cell division was the key factor in the eventual loss of the knockdown effect [45, 46]. Taken together, the optimization of culture conditions, which allows cells to take up as much siRNA as possible, may be the key to obtaining the best knockdown results.

## Supplementary data

Supplementary data are available at *Biology Methods and Protocols* online.

## Supplementary Material

bpac011_Supplementary_Data
