# Supplementary figures and images for: Establishing an effective gene knockdown system using cultured cells of the model fish medaka (Oryzias latipes)
Source: Biol Methods Protoc. 2022 May 17;7(1):bpac011. doi: 10.1093/biomethods/bpac011 (PMC9171500; doi:10.1093/biomethods/bpac011)

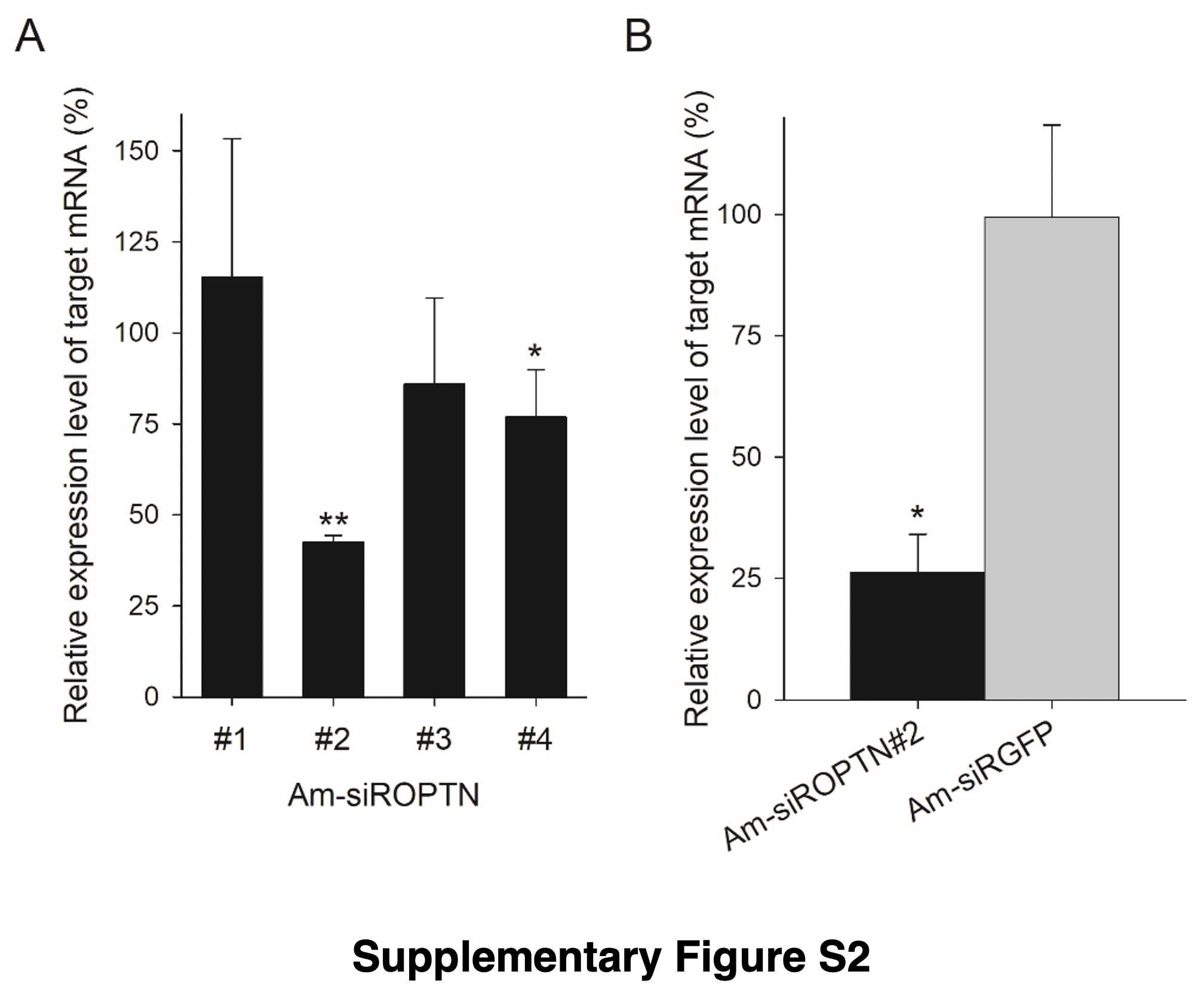

Supplement: bpac011_Supplementary_Data [file bpac011_supplementary_data.zip › Fig. S2 (Zenke and Okinaka) (1).jpg]

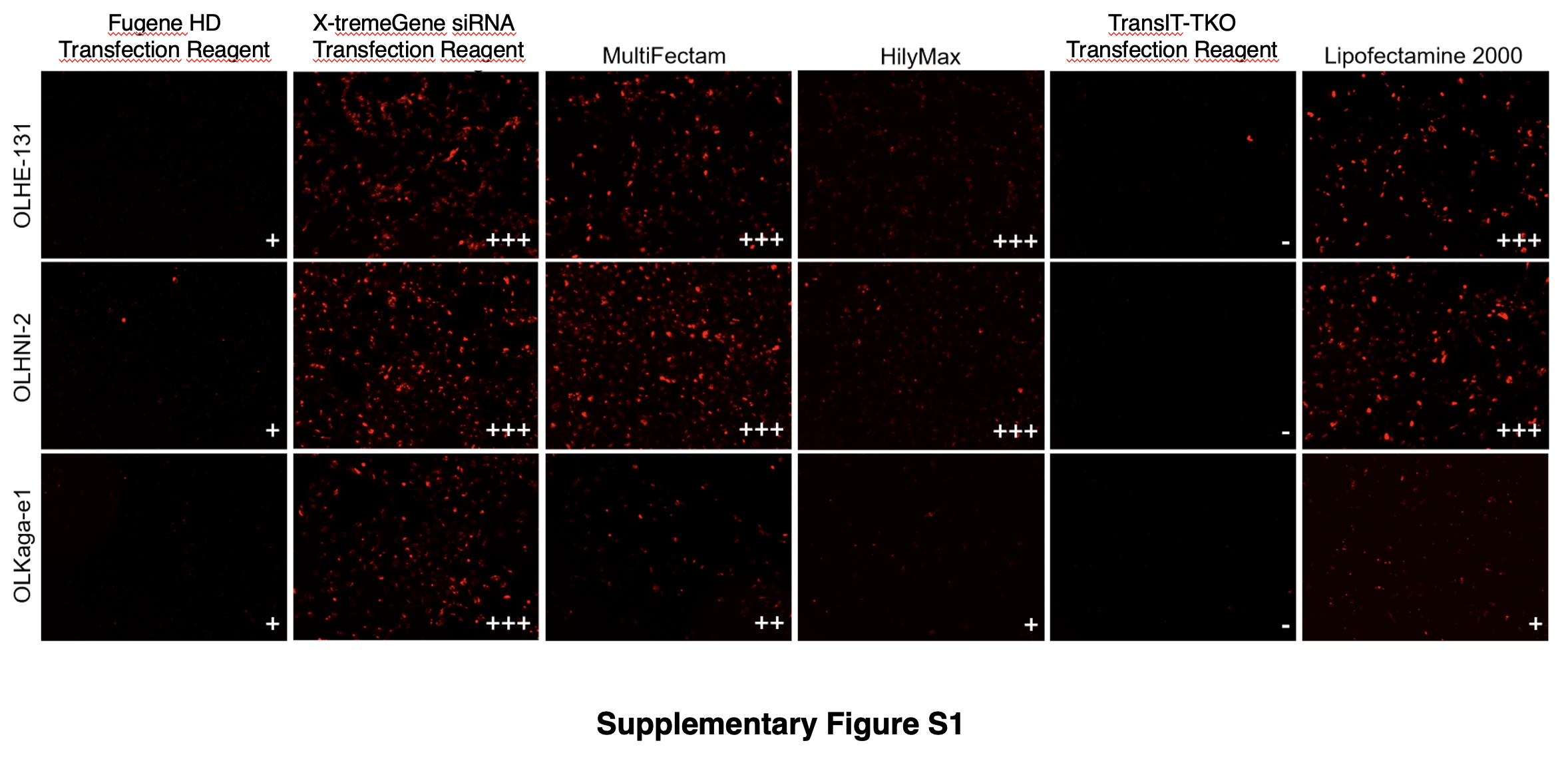

Supplement: bpac011_Supplementary_Data [file bpac011_supplementary_data.zip › Fig. S1 (Zenke and Okinaka) (1).jpg]
